# Desperately Seeking Verity: Causes of Death in the Dialysis Population

**DOI:** 10.34067/KID.0000000730

**Published:** 2025-03-27

**Authors:** Daniel L. Landry, Gregory L. Braden

**Affiliations:** Baystate Medical Center, Springfield, Massachusetts

**Keywords:** cardiovascular events, dialysis, ESKD

Patients with ESKD receiving dialysis die of different variations of cardiovascular disease when compared with the general population. For instance, registry data and prospective studies have shown that sudden cardiac death (SCD) is a much more common cause of cardiovascular death than the more commonly recognized atherosclerotic plaque rupture events suffered by the non-ESKD population.^1,2^ To monitor the causes of death (CODs) in patients with ESKD, the US government established federally mandated documentation regarding CODs by the Health Care Financing Administration (formed in 1977 and now known as the Centers for Medicare & Medicaid Services [CMS]). Statistical analysis of the United States Renal Data System (USRDS) annual data report has elucidated the most frequent COD in patients with ESKD and is built off of CMS form 2746 (CMS-2746) claims data, which contains 77 unique options for physicians to mark as the cause of death, which are then generalized into broader COD categories (SCD, cardiovascular, infection, unknown, and others). To dig deeper, however, one must also go beyond SCD and non-SCD to identify distinct risk phenotypes that align with different CODs that commonly afflict the ESKD population. In addition, there also remains the issue that with any database comes the readily apparent recognition that “poor data in equates to poor data out.”

In this issue of *Kidney360*, Tran *et al.* have approached the difficult task of identifying CODs by comprehensive analyses of the electronic health record data 30 days before death from patients with ESKD cared for by a single dialysis provider (Dialysis Clinics, Inc.) between 2003 and 2016.^3^ The data were linked to USRDS clinical files in these patients to identify patients by distinct CODs. They used machine learning with XgBoost, least absolute shrinkage and selection operator regression-based analyses, and ridge analysis methods for the first time ever to define death subgroups in patients with ESKD. They also performed a nested case–control group temporally matched from living Dialysis Clinics, Inc. patients during the same period. The authors concluded that most clinical risk markers for death were similar among patients with ESKD who died of SCD, non-SCD cardiovascular causes, infection, or other causes and in the control group. The addition of data from the National Death Index (NDI) added no benefit to their data analysis.

A review of the data in the paper by Tran et al finds that whether patients died of SCD, non-SCD cardiovascular, infectious, or other disease causes, the comorbidities of each of the four groups were remarkably similar and often more frequent than the comorbidities usually reported in the USRDS registry. For example, the average frequency of comorbidities in their study are as follows: atherosclerotic heart disease 80%, congestive heart failure 85%, cerebrovascular accident 60%, peripheral vascular disease 80%, and chronic obstructive pulmonary disease 60%. These frequencies were also almost exactly similar in their control group. By contrast, the aggregate frequencies of these comorbidities from the 2022 USRDS registry were much lower: atherosclerotic heart disease 46%, congestive heart failure 48%, cerebrovascular accident 12%, peripheral vascular disease 45%, and chronic obstructive pulmonary disease 12%.^4^ It is unclear why the frequencies of comorbidities were so high in all the groups in the study by Tran et al. It is possible that coding errors of comorbidities could have occurred, skewing the data analyses. However, it seems clear that with the frequencies being so high and similar in the control groups, even the best statistical methods used by these investigators, would not have allowed discrimination between death subgroups.

Several studies of the COD in patients with ESKD have compared the CODs on the CMS-2746 form from the USRDS registry to the cause listed on the patients' death certificate in the NDI established by the National Center of Health Care vital statistics. Indeed, the same current investigators have previously identified discrepancies in SCD occurring in the USRDS (42%) versus the NDI (22%) with only a 42% agreement (k=0.23), when comparing USRDS with the NDI COD for 39,507 patients on incident hemodialysis (HEMO) from 2003 to 2009.^5^ Similar results were found in a Kaiser Permanente study comparing the CODs in patients with ESKD studied from 2007 to 2016 between USRDS and the Kaiser death registry. The CODs only matched 36.4%.^6^ The kappa statistic between the two death classification systems using Cohen's weight had a 95% confidence interval of only 0.2. Thus, some of the inability to define markers for death groups in ESKD seems to be impeded by incomplete or inaccurate data sources to input for analysis.

By contrast, smaller studies of death in the ESKD population have used adjudicated methods to improve the definitive COD in these patients. The HEMO Study Group^7^ used a trained HEMO study outcome committee of physicians who reviewed deceased ESKD patients' records in detail. Interim analysis of their mortality data was compared with CMS-2746. Among the 220 deaths coded by both classification systems, the kappa statistic between the two death classification systems ranged from only 0.12 for congestive heart failure to 0.95 for malignancy, raising concerns with the accurate labeling of CODs secondary to either cardiovascular diseases or unknown causes by the form CMS-2746.

Pun *et al.* used a US Gambro (DaVita) outpatient dialysis population to analyze 363 patients with witnessed SCD and retrospectively examined the performance characteristics of a newer method of SCD classification when added to USRDS data.^8^ The sensitivity to SCD defined by death notification forms and SCD defined using additional administrative sources was compared in addition to clinical data recorded around the time of death. The authors found the refined definition significantly improved proper identification of SCD from 70.8% USRDS alone to 83.8% of witnessed SCD events (*P* < 0.001). The authors found that most misidentified SCD CODs were labeled as myocardial infarction, hyperkalemia, sepsis, malignancy, or unknown cause.

Another small single-center study of risk factors of SCD used a team of physicians, dialysis nurses, and social workers familiar with their patients to review all deaths during a Renal Palliative Care initiative funded by the Robert Wood Johnson Foundation.^9^ All aspects of the deaths in these patients were carefully reviewed before the CMS-2746 was submitted. In addition, all demographic, clinical, dialysis, and laboratory data were collected at the time of death and for up to 3 months before death occurred. In this study, numerous risk factors of SCD were identified on univariate analysis, and most importantly, multivariate analysis revealed that elemental calcium intake >3000 gm/d, prolonged QT interval, and bundle branch block on electrocardiogram were associated with SCD.

We applaud Tran and coauthors in their effort to better understand factors relating to the CODs in patients with ESKD by using for the first time novel methods of data analysis, including machine learning with XgBoost, least absolute shrinkage and selection operator linear regression, and ridge analysis models, to determine definitive risk factors of SCD, non-SCD cardiovascular death, infectious causes, and other CODs as outlined by CMS-2746. The results seem to cast a light on the accuracy of data supplied to the USRDS database through CMS-2746 and the NDI. Efforts to improve the COD description (particularly in the setting of cardiac cause) would be of great benefit for the purposes of better understanding the why and how of ESKD mortality. In the meantime, perhaps future studies with more carefully adjudicated methods of COD using comprehensive prospective data collection over an extended period will be necessary to better characterize the markers and risk factors of the different CODs in patients with ESKD. Until that time comes, we may be left with inaccurate data leading to inaccurate analysis and (even worse) inaccurate decision making when it comes to addressing the CODs in our dialysis community.
